# Reversals and limitations on high-intensity, life-sustaining treatments

**DOI:** 10.1371/journal.pone.0190569

**Published:** 2018-02-28

**Authors:** Gustavo Chavez, Ilana B. Richman, Rajani Kaimal, Jason Bentley, Lee Ann Yasukawa, Russ B. Altman, Vyjeyanthi S. Periyakoil, Jonathan H. Chen

**Affiliations:** 1 Stanford University School of Medicine, Stanford, California, United States of America; 2 Center for Outcomes Research & Evaluation, Yale Medicine, New Haven, CT, United States of America; 3 Stanford Quantitative Sciences Unit, Stanford, California, United States of America; 4 Stanford Center for Clinical Informatics, Stanford, California, United States of America; 5 Department of Bioengineering, Stanford University School of Medicine, Stanford, California, United States of America; 6 VA Palo Alto Health Care System, Palo Alto, California, United States of America; 7 Department of Medicine, Stanford University School of Medicine, Stanford, California, United States of America; 8 Center for Biomedical Informatics Research and Division of Hospital Medicine, Department of Medicine, Stanford University School of Medicine, Stanford, CA, United States of America; University of Florida, UNITED STATES

## Abstract

**Importance:**

Critically ill patients often receive high-intensity life sustaining treatments (LST) in the intensive care unit (ICU), although they can be ineffective and eventually undesired. Determining the risk factors associated with reversals in LST goals can improve patient and provider appreciation for the natural history and epidemiology of critical care and inform decision making around the (continued) use of LSTs.

**Methods:**

This is a single institution retrospective cohort study of patients receiving life sustaining treatment in an academic tertiary hospital from 2009 to 2013. Deidentified patient electronic medical record data was collected via the clinical data warehouse to study the outcomes of treatment limiting Comfort Care and do-not-resuscitate (DNR) orders. Extended multivariable Cox regression models were used to estimate the association of patient and clinical factors with subsequent treatment limiting orders.

**Results:**

10,157 patients received life-sustaining treatment while initially Full Code (allowing all resuscitative measures). Of these, 770 (8.0%) transitioned to Comfort Care (with discontinuation of any life-sustaining treatments) while 1,669 (16%) patients received new DNR orders that reflect preferences to limit further life-sustaining treatment options. Patients who were older (Hazard Ratio(HR) 1.37 [95% CI 1.28–1.47] per decade), with cerebrovascular disease (HR 2.18 [95% CI 1.69–2.81]), treated by the Medical ICU (HR 1.92 [95% CI 1.49–2.49]) and Hematology-Oncology (HR 1.87 [95% CI 1.27–2.74]) services, receiving vasoactive infusions (HR 1.76 [95% CI 1.28, 2.43]) or continuous renal replacement (HR 1.83 [95% CI 1.34, 2.48]) were more likely to transition to Comfort Care. Any new DNR orders were more likely for patients who were older (HR 1.43 [95% CI 1.38–1.48] per decade), female (HR 1.30 [95% CI 1.17–1.44]), with cerebrovascular disease (HR 1.45 [95% CI 1.25–1.67]) or metastatic solid cancers (HR 1.92 [95% CI 1.48–2.49]), or treated by Medical ICU (HR 1.63 [95% CI 1.42–1.86]), Hematology-Oncology (HR 1.63 [95% CI 1.33–1.98]) and Cardiac Care Unit-Heart Failure (HR 1.41 [95% CI 1.15–1.72]).

**Conclusion:**

Decisions to reverse or limit treatment goals occurs after more than 1 in 13 trials of LST, and is associated with older female patients, receiving non-ventilator forms of LST, cerebrovascular disease, and treatment by certain medical specialty services.

## Introduction

Advances in medical technology have generated multiple sophisticated life sustaining treatment (LST) options. These capabilities have undoubtedly saved or at least prolonged many patient lives, but their development may not always be matched by the prudence to apply them.

Effective decisions for LSTs and cardiopulmonary resuscitation are challenging when underuse can represent a life-threatening patient safety hazard[1] while overuse can have its own severe adverse consequences. Inappropriate decisions to withhold life-sustaining treatments have severe consequences,[2,3] and there is risk of patients labeled with “do not resuscitate” orders being misinterpreted to receive generally less aggressive care that they may benefit from. For this study, we focus on the complementary scenario. When both health professionals and the public systematically overestimate the efficacy of LSTs,[4] critically ill patients may receive burdensome high intensity treatments that do not effectively improve their quality or quantity of life.[5–7] Patients receiving LSTs who subsequently reverse or limit their goals of care with physician orders for Comfort Care or DNR can improve our understanding of the challenging LST decision making process and the effects of their application.[8–13]

Prior studies demonstrate differences in DNR orders based on patient characteristics, family, providers, and institutions.[10,12–24] Specifically, advanced age, female gender, and White race are associated with greater limitations, like DNR orders,[10,13,19,23] while physician projection of future mortality and recovery, age, patient wishes, severity of illness, and number of comorbidities are associated with withdrawal of life support.[25] Such differences can occur despite established guidelines, in part due to medical providers’ poor understanding of advance care planning documents such as living wills or physician orders for life sustaining treatments (POLST).[26,27]

Prior surveys suggest that physicians of different specialties have varying recommendations for end-of-life decision making.[14,15,28] Determining the factors associated with reversals in LST goals can improve patient and provider appreciation for the natural history and epidemiology of critical care to inform decision making on (continued) use of LSTs. In this study, we focused specifically on patients where life-sustaining critical care interventions were initiated, then had a subsequent reversal or limitation during the same hospitalization. We also evaluated the relevance of various modalities of life support and the potential variation in different specialty treatment teams in the decision-making process. The study was exempted as non-human subjects research by the Stanford IRB.

## Methodology

### Study population

Deidentified patient data from Stanford University Hospital from 2009 to 2013 were collected via the STRIDE clinical data warehouse.[29] Patients were included if they received any life-sustaining critical care interventions as recorded in electronic medical records; mechanical ventilation, vasoactive infusions (dopamine, norepinephrine, epinephrine, dobutamine, vasopressin, phenylephrine), or continuous renal replacement therapy (CRRT). The structured data includes patient encounters from their initial life support order until hospital discharge. Patient demographics, comorbidities, laboratory results, vital signs, and primary medical team assignments were also included.

### Study outcomes

In the Stanford Health Care system, a patient’s preferences regarding LST are interpreted and summarized in a code status order electronically signed by an attending physician. Code status options include: Full Code, Partial Code, DNR, and Comfort Care. The default Full Code status indicates no limits on resuscitative measures. Partial Code includes specific limitations that are generally less restrictive than a complete DNR order, such as no intubation, chest compressions, or defibrillation. DNR indicates to allow natural death without resuscitative measures in the event of cardiopulmonary arrest (pulseless and apneic). Comfort Care is essentially the extreme end of DNR orders, indicating symptom-oriented treatments only, removing interventions only intended to prolong life. This typically occurs when death is imminently expected as any current life-sustaining therapies that are inconsistent with an active Comfort Care order are discontinued.

To study reversals in LST, we identified patients who were Full Code as of life support initiation and followed them from this point until hospital discharge, death, or change to Partial Code, DNR or Comfort Care. The primary study outcome was change to Comfort Care status. As a secondary (but more prevalent) outcome, we assessed for change to “Any DNR” code status (Partial Code, DNR, or Comfort Care) that may not reflect a complete reversal, but a limitation on subsequent LST.

### Covariates

We assessed patient and clinical factors for association with limiting LST. Patient factors included race/ethnicity, age, gender, insurance status (Self-pay or not), and socioeconomic status. Socioeconomic status was approximated by median household income of their home ZIP code from 2013 census data.[30] Patients’ Charlson comorbidities were determined based on International Classification of Diseases 9th edition (ICD-9) diagnosis codes in patient problem lists and admission diagnosis.[31] We used binary indicators for each of the 16 comorbidities. To adjust for acute severity of illness that could dynamically effect decision making, we included daily laboratory results and vital signs based on components of the APACHE[32] and SAPS[33] scores. These included temperature, systolic blood pressure, pulse, respiratory rate, partial pressure of oxygen (pO_2_(a)), arterial pH (pHa), sodium, potassium, creatinine, blood urea nitrogen (BUN), total bilirubin, bicarbonate, hematocrit, white blood cells and Glasgow coma scale score (GCS). The life sustaining treatment modalities used (i.e., mechanical ventilation, vasoactive infusion, or CRRT) were also noted. Lastly, we included binary indicators for having been treated by different primary medical teams (e.g., cardiology, medicine, neurology, hematology-oncology, critical care, surgery, transplant, or trauma). Each patient can have accumulated multiple treatment teams over the course of their stay.

### Statistical analysis

We described the study population with contingency tables. We evaluated the association of baseline (patient demographics and comorbidities) and time-varying (primary medical team, life sustaining treatment, vital signs and lab test results) characteristics on change to Any DNR and change to Comfort Care status using extended multivariable Cox proportional hazards regression models. Vital sign and lab results had missing values due to being intermittently assessed (i.e. are not necessarily required every day) or not being required if a patient is healthy or on life sustaining treatment (e.g. CRRT induces normative potassium levels). As such, the data is likely to be missing not at random. To deal with this we used a combined single imputation approach as follows: for each vital sign or lab test from the first available value, last observation carried forward (LOCF) was used to fill in missing values. For observations missing prior to the first available value were filled in using the normative healthy range for each vital sign or test based on ranges used in the APACHE and SAPS scores as follows: Temperature 98.6F, Systolic Blood Pressure 110mm Hg, Pulse 80bpm, Respiratory rate 16breaths/min, pO2(a) 95mmHg, pHa 7.4, Sodium 145mEq/L, Potassium 4.5mEq/L, Creatinine 0.8mg/dl, Hematocrit 42%, White blood cells 8 × 10^9^/L, Glasgow Coma Scale score 15, Blood urea nitrogen 15mg/dl, Total bilirubin 1mg/dl, Bicarbonate 24mEq/L. To assess the sensitivity of results to different healthy normative values for initial vital sign and lab test measures, we estimated three additional Cox models for each study outcome as follows: without adjustment for vital sign and lab test measures at all (essentially ignoring acute severity of illness); using minimum healthy range values, and using maximum healthy range values. Notably, vital sign and lab test values may be highly correlated with receiving life support (e.g., blood pressure with vasoactive infusions or respiratory rate with ventilation). Thus, three further Cox models were fit to assess the sensitivity of the associations for life support covariates (CRRT, vasoactive infusion, ventilation) to vital sign and lab test imputation. For each study outcome, three models (using LOCF and normative healthy values) assessed each life support covariate in turn while excluding those vital signs or lab test results that are expected to be normative due to the life support mechanism itself. These models were as follows: for CRRT sodium, potassium, creatinine, blood urea nitrogen, and bicarbonate were excluded; for vasoactive infusion pulse and systolic blood pressure were excluded; and for ventilation respiratory rate, pO2(a), and PHA were excluded.

For each model we applied a Bonferroni correction when assessing that significance of covariates. As each model contained 50 covariates and assuming a desired family-wise alpha of 0.05, the threshold for significance is 0.05/51 = 0.001. Data management was done with Python 2.7 and SAS 9.3, while analyses were conducted with R version 3.3.2

## Results

### Baseline characteristics

**Fig 1:**
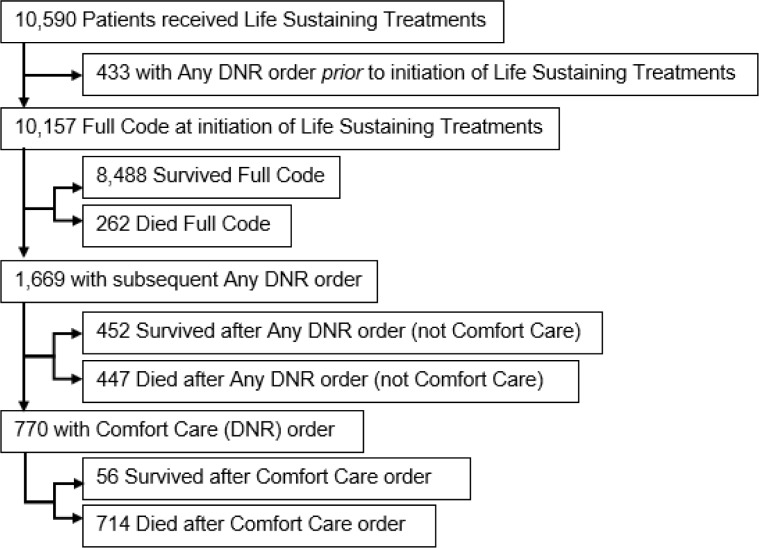
Flowchart of patients receiving life sustaining treatments, code status orders, and in hospital mortality.

During the study period, 10,590 subjects received LSTs. Of these, 433 already had Any DNR order at the initiation of LST, likely receiving them for critical care support before overt cardiopulmonary arrest (e.g., vasopressors for hypotension or mechanical ventilation for hypoxia). This leaves 10,157 patients in the study cohort who were Full Code at the entry point of initiating LST, with baseline characteristics summarized in Table 1. After initiation of LST, 1,669 (16%) subsequently had Any DNR order, 770 (8%) had Comfort Care orders, and 1,423 (14%) died, as summarized in Table a in S1 File and Fig 1.

**Table 1:** Summary of patient and clinical characteristics (N = 10,157).

Baseline Characteristic	Median [IQR] or Number (Percent)
Age (years)	62.2 [49.9–73.6]
Length of Stay (days)	8.0 [5.0–15.0]
Household Income Estimate	$70001 [52001–95001]
Insured	10035 (99%)
Female	4,257 (42%)
Race/Ethnicity	
Non-Hispanic White	5,664 (56%)
Hispanic/Latino	1,512 (15%)
Native American	35 (0.34%)
African-American	492 (5%)
Pacific Islander	149 (1%)
Asian	1269 (12%)
Other	592 (6%)
Unknown	444 (4%)
Charlson Comorbidities	
Cerebrovascular Disease	725 (7%)
Congestive Heart Failure	768 (8%)
COPD	472 (5%)
Dementia	8 (0.1%)
Diabetes without Chronic Complications	590 (6%)
Diabetes with Chronic Complications	97 (1%)
Hemiplegia or Paraplegia	45 (0.4%)
Malignancy	1518 (15%)
Metastatic Solid Tumor	205 (2%)
Myocardial Infarction	27 (0.3%)
Mild Liver Disease	414 (4%)
Moderate or Severe Liver Disease	230 (2%)
Peptic Ulcer	37 (0.4%)
Peripheral Vascular Disease	682 (7%)
Renal Disease	520 (5%)
Rheumatologic Disease	98 (1%)
Medical Teams	
Cardiology	913 (9%)
Cardiac Care Unit (CCU)	751 (7%)
Cardiac Care Unit-Heart Failure (CCU(HF))	370 (4%)
Cardiovascular ICU (CVICU)	280 (3%)
Hematology-Oncology	410 (4%)
Medicine	1179 (12%)
Medical ICU	718 (7%)
Neurology	117 (1%)
Surgical ICU	506 (5%)
Surgery Specialty	1078 (11%)
Transplant	530 (5%)
Trauma	234 (2%)
Life-Sustaining Treatment Usage at initial baseline*	
CRRT	288 (3%)
Vasoactive	6,727 (66%)
Ventilator	7,414 (73%)
Life–Sustaining Treatment usage at any time of stay*	
CRRT	691 (7%)
Vasoactive	7,442 (73%)
Ventilator	7,932 (78%)
Comfort Care	770 (8%)
Any DNR	1,669 (16%)
Died during study period	1,423 (14%)
Died with Comfort Care orders	714 (50%)
Died with Any DNR	1,161 (82%)

*Not Mutually exclusive categories

### Patient demographics

Comfort Care orders were more likely in older patients (HR 1.37 [95% CI 1.28–1.47] per decade). Any DNR orders were more likely with older patients (HR 1.43 [95% CI 1.38–1.48] per decade) and female patients (HR 1.3 [95% CI 1.17–1.44]).

No significant racial disparities were detected for Any DNR orders in multivariable analysis relative to the most prevalent reference group (Non-Hispanic White).

### Comorbidity categories

Comfort Care orders were more likely in patients suffering from Cerebrovascular Disease (HR 2.18 [95% CI 1.69–2.81]) or those who have had a Myocardial Infarction (MI), (HR 4.43 [95% CI 1.85–10.62]) while Any DNR orders were more likely for those with Cerebrovascular Disease (HR 1.45 [95% CI 1.25–1.67]), Metastatic Malignancy (HR 1.92 [95% CI 1.48–2.49]), or other Malignancy (HR 1.33 [95% CI 1.15–1.54]).

### Life-sustaining treatment modality

Comfort Care orders were more likely for patients receiving vasoactive infusions (HR 1.76 [95% CI 1.28–2.43]) and continuous renal replacement therapy (HR 1.83 [95% CI 1.34–2.48]). Any DNR was similarly more likely for patients receiving vasoactive infusions (HR 1.55 [95% CI 1.31–1.84]) and continuous renal replacement therapy (HR 1.43 [95% CI 1.2–1.7]). Mechanical ventilation was not significantly associated with either Any DNR or Comfort Care orders.

### Treatment team specialty

Patients treated by the Medical ICU (HR 1.92 [95% CI 1.49–2.49]) and Hematology-Oncology (HR 1.87 [95% CI 1.27–2.74]) appeared were more likely to transition to Comfort Care, though the Hematology-Oncology association did not meet our stricter threshold for significance.

Patients treated by the Medical (HR 1.63 [95% CI 1.42–1.86]), Hematology-Oncology service (HR 1.63 [95% CI 1.33–1.98]), and Cardiac Care Unit-Heart Failure service (HR 1.41 [95% CI 1.15–1.72]) were more likely to change to Any DNR, while those treated by the Cardiovascular ICU (HR 0.32 [95% CI 0.25–0.41]), and Surgical services (HR 0.74 [95% CI 0.63–0.87]) were less likely.

**Fig 2:**
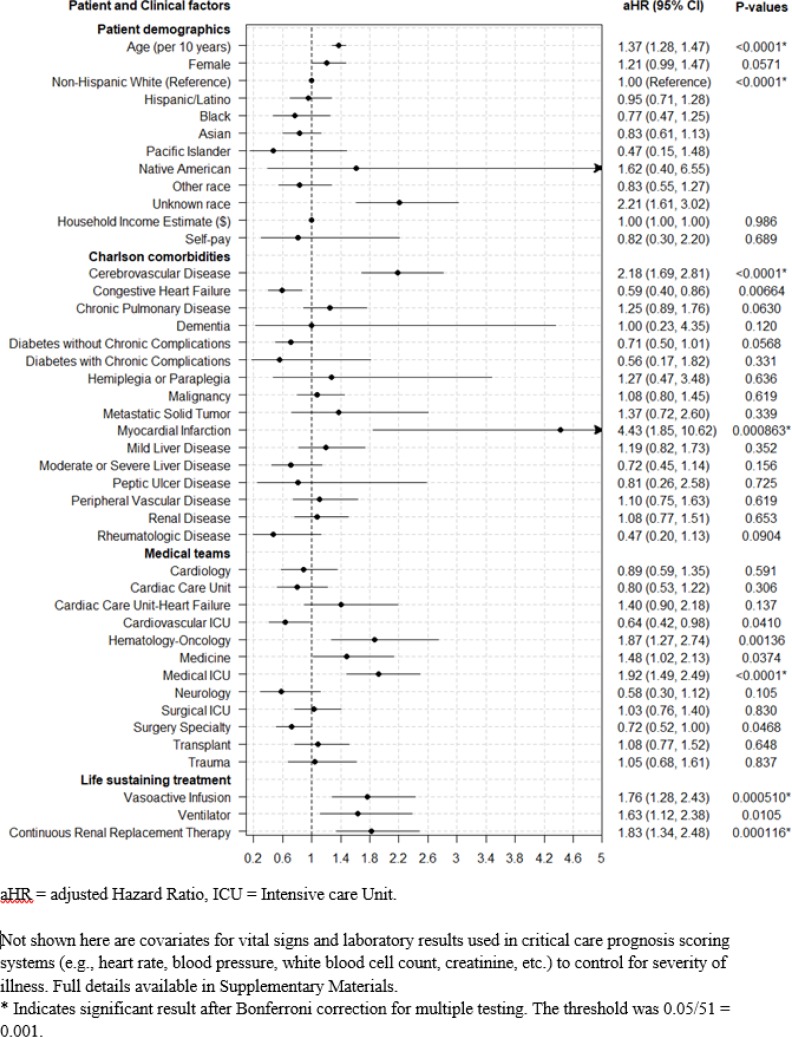
Predictors of ICU patients transitioning to Comfort Care.

**Fig 3:**
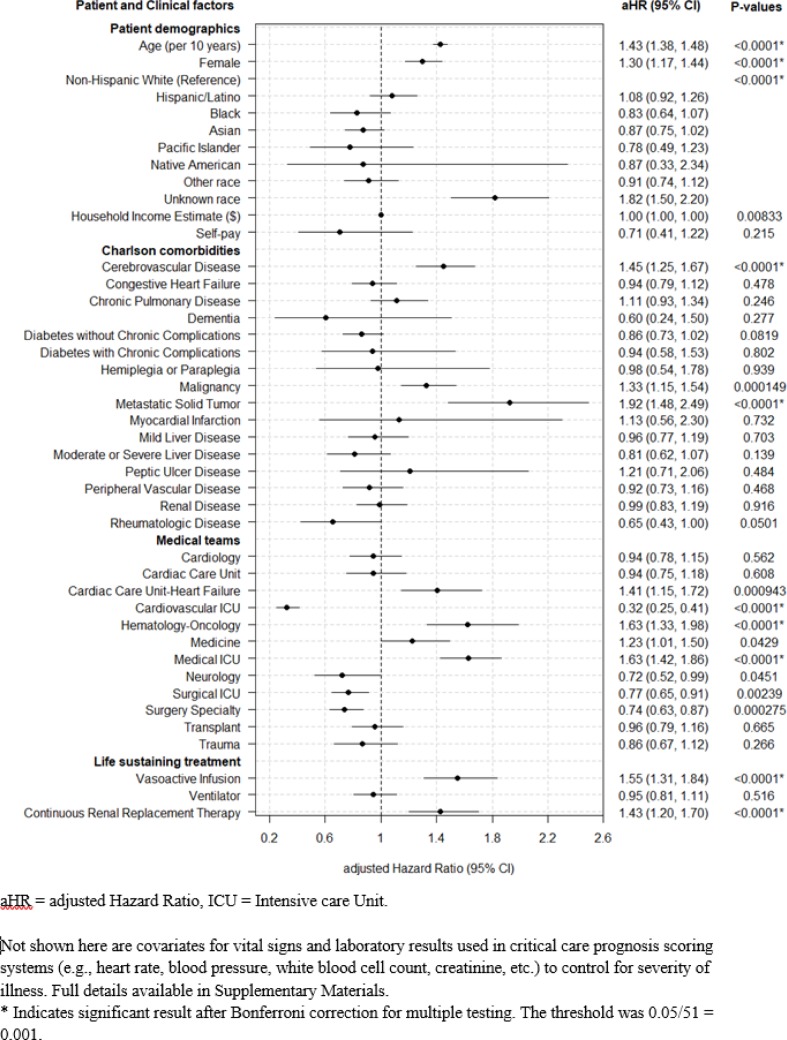
Predictors of ICU transitioning to any DNR (do-not-resuscitate).

Primary results are summarized in Figs 2 and 3. Supplementary Materials includes results for multiple additional variations of the Cox model for sensitivity analysis with respect to different data imputation strategies and inclusion vs. exclusion of different vital signs and lab result indicators of severity of illness.

## Discussion

Most inpatients entering life-sustaining critical care interventions were Full Code, which may reflect purposeful choices or limited prior advance care planning discussions (e.g., “default” selection that was not pointedly discussed with patients). Only 4.1% of patients had Any DNR order at initiation of LST. Among the Full Code patients, 8.0% subsequently had Comfort Care orders and 16% subsequently had Any DNR order. Of the 1,423 patients who died in the hospital, 714 (50%) died with Comfort Care orders and 1,161 (82%) died with DNR orders.

Prior literature suggests racial and cultural disparities in decision making,[10,19,21,23] though we found no significant differences for Hispanic, African American, Pacific Islander, or Native American patients changing to Comfort Care or Any DNR *after* controlling for other patient and clinical factors. The lack of significant disparities among racial minority groups suggests that differences are due to alternative characteristics, such as comorbidity, severity of illness, treatment modality, and team specialty. Similar to prior studies,[10,13,19,23] we found that advancing age and female gender are associated with decisions to limit life sustaining treatments, even after adjusting for other factors. Ongoing research and discussion are warranted to properly interpret and apply decision making in these cases to ensure both patient safety and informed consent.

We found that the modality of life-sustaining critical care interventions had varying associations with Comfort Care or Any DNR orders. Patients on either continuous renal replacement therapy or vasoactive infusions were more likely to receive Any DNR order to limit further escalation of aggressive life support interventions. Mechanical ventilation was not significantly associated with changing code status. This may be because patients are less willing to transition, or they have a physical barrier (intubation and sedation) to expressing revised goals of care. In the latter case, decision making responsibility then goes to the patient’s surrogate (typically family), who may be more hesitant to advocate reversals and limitations than the patient themselves if not previously addressed in documents like living wills. Alternative processes to best represent patient preferences rather than family member’s best guesses are thus an additional important area of development.[1]

Notably, we found that the treatment teams involved in patient care were associated with Comfort Care and Any DNR. Patients transferred to the Intensive Care Unit from Hematology and Oncology services were more likely to have Any DNR orders, while patients from Surgical, Cardiovascular and Cardiac Intensive Care Unit services were less likely. Decision variation is expected when different primary specialty teams reflect patients with different disease states (e.g. cancer), corresponding prognosis, and acceptable treatment goals (e.g. elective surgery). Yet, these variations are observed even after adjusting for patient comorbidities, admission diagnosis, and severity of illness indicators, which raises questions about differing practice patterns within various medical subspecialists. Clinicians and patients have different perspectives or abilities to recognize conditions amenable to intervention. For example, we may recognize metastatic cancer as an intractable terminable disease while believing there are always more interventions to salvage patients with cardiac and surgical problems. At the same time, evidence suggests that in some (surgical) settings, prematurely labeling patients “DNR” may result in worse outcomes perhaps from unintended “failure to rescue.”[34,35] This study alone cannot judge whether any of the observed practices are “appropriate” or not, but does illustrate that differences exist.

Supplementary Materials review the results of multiple sensitivity analyses with respect to different data imputation strategies and inclusion vs. exclusion of different vital signs and lab result indicators of severity of illness. The overall pattern of significant findings remained stable across different model variations, except for use of mechanical ventilation. In models excluding vital signs and laboratory results, mechanical ventilation is positively associated with subsequent DNR orders. This likely just reflects mechanical ventilation as a proxy for severity of illness as our primary model that included markers for acute illness did not show a significant association.

Limitations of this study include that data was collected from a single institution, which can restrict generalizability. Prior studies demonstrated that do not resuscitate physician orders vary according by region and are less prevalent in teaching hospitals than community hospitals.[23] The patient community surrounding the study institution generally represents a wealthier, more educated, and ethnically diverse population.[36,37] Our results identify patient and clinical factors associated with Comfort Care and Any DNR orders, but do not necessarily represent causal relationships. Similarly, given this retrospective study of structured electronic medical record data, additional contextual information such as living wills,[38] physician orders for life sustaining treatment,[39] and documentation of physician-family meetings were unavailable to provide qualitative rationale for the decisions in individual cases. Our study assumes that DNR orders electronically signed by attending physicians are “correct,” but does not account for the possibility of physicians misinterpreting patient preferences for life-sustaining interventions.[1]

While Comfort Care orders are a relatively explicit proxy for reversing LST decisions, Any DNR order after LST initiation may still be consistent with initial LST treatment goals (e.g., starting on vasopressors for low blood pressure, but later activating a DNR order to decline mechanical ventilation while still accepting ongoing vasopressors). Even in the case of subsequent Comfort Care orders, it is worth reflecting that while this may ultimately be a “reversal” of goals of care for ineffective LST, this does NOT entail that the decision to initiate LST was a “mistake.” As with all medical treatments, many trials of therapy do not ultimately produce a benefit. The initial decision to offer the therapy should be judged based only on information available at that moment, which is perfectly valid when we credibly expect a potential benefit that outweighs risks and harms.

What the results of this study can do is inform discussions around critical care decisions, similar to other prognostic risk scoring systems. Estimating patient risks for changing preferences can shift the balance of risks, benefits, and harms that are acceptable to patients when proceeding with trials of potentially burdensome, but life sustaining, treatments. Clinicians and health systems should take interest in identifying areas of care variation and in the characteristics, that may reflect critically ill patients who are more receptive to goals of care discussions, to help us deploy supportive and palliative interventions as early as possible in appropriate cases.

In summary, we found that Comfort Care and Any DNR orders subsequently occur for more than 1 in 13 patients who received life sustaining therapy, and are more common among older women. Patients on different forms of LST (e.g., continuous renal replacement and vasopressors) were more likely to reverse treatment compared to those on ventilator support. Different medical treatment team subspecialties are associated with varying resuscitation status. Awareness of these predictive factors can help inform complex decision-making processes to mitigate the risks of both underuse and overuse of critical care interventions.

## Supporting information

S1 File Table a and b. Frequency and percent of Non-missing values for vital signs and lab tests. Frequency and percent of Non-missing values for vital signs and lab tests after missing values were replaced using last value carried forward.
Table c. Vital sign and lab test healthy ranges and correspondence with medical support.
Table d. Comparison of coefficients from primary and sensitivity analyses for Comfort Care Comparison of coefficients from primary and sensitivity analyses for Any DNR.
Fig a. Example pseudo-record.
Fig b. Comparison of coefficients from primary and sensitivity analyses for comfort care.
Fig c. Comparison of coefficients from primary and sensitivity analyses for Any DNR.
(DOCX)
