# Supplementary material for: Reversals and limitations on high-intensity, life-sustaining treatments
Source: PLoS One. 2018 Feb 28;13(2):e0190569. doi: 10.1371/journal.pone.0190569 (PMC5830043; doi:10.1371/journal.pone.0190569)
Supplement: S1 File — Table a and b. Frequency and percent of Non-missing values for vital signs and lab tests. Frequency and percent of Non-missing values for vital signs and lab tests after missing values were replaced using last value carried forward. Table c. Vital sign and lab test healthy ranges and correspondence with medical support. Table d. Comparison of coefficients from primary and sensitivity analyses for Comfort Care Comparison of coefficients from primary and sensitivity analyses for Any DNR. Fig a. Example pseudo-record. Fig b. Comparison of coefficients from primary and sensitivity analyses for comfort care. Fig c. Comparison of coefficients from primary and sensitivity analyses for Any DNR. (DOCX) [file pone.0190569.s001.docx]

**Supplementary: Statistical Methods**

*Missing vital sign and lab test information*

Vital sign and lab test information (temperature, systolic blood pressure, pulse, respiratory rate, pO2(a), pHa, sodium, potassium, creatinine, hematocrit, white blood cells, Glasgow Coma Scale score, blood urea nitrogen, total bilirubin, bicarbonate) was missing because observations/tests are only conducted intermittently and not done every day. Furthermore, when vital sign and lab test information is missing, it is because the patient is healthy or receiving medical support that induces normative values (e.g. continuous renal replacement therapy [CRRT] and sodium, potassium, creatinine, blood urea nitrogen, and bicarbonate levels), and so measurement is unnecessary. This implies that the missingness of vital sign and lab test information depends upon its value, and so could be considered as missing not at random (MNAR). To overcome this, missing values were replaced using last value carried forward (LOCF).**Supplementary Table a** details the number and percent of measures recorded each day for the first four days and **Supplementary table b** details the number and percent of measures after missing values were replaced using last value carried forward To deal with the fact that many patients did not have a first vital sign or lab test measurement until a few days into their hospital stay, mean values according to the normal health range were used. **Supplementary Figure a** provides an example of a pseudo-record structure for a patient with any DNR and **Supplementary Table c** details the mean healthy values used for vital signs and lab tests. The extended Cox model using this approach is presented as the primary analysis in the manuscript.

Due to assuming mean values for the first few days of patients stays for missing vital sign and lab test information, we conducted sensitivity analysis. We considered three additional Cox models as follows: (i) excluding vital signs and lab tests (i.e. covariates with missing information), (ii) using minimum healthy range values for vital sign and lab test information for days prior to the first measurement and LOCF thereafter, and (iii) using maximum healthy range values for vital sign and lab test information for days prior to the first measurement and LOCF thereafter. **Supplementary Table c** details the minimum and maximum healthy values used for vital signs and lab tests.

An additional complication arising from this approach is that receiving medical support induces normative values in selected vital sign and lab test measurements and so are co-linear to an extent with the covariates indicating medical support (CRRT, vasoactive infusion, ventilation). **Supplementary Table c** details the correspondence between vital signs, lab tests and the covariates for medical support used in this study. To investigate the sensitivity of the associations for each of these covariates (CRRT, vasoactive infusion, ventilation) to the approach used for missing vital sign and lab test information we fitted an additional three Cox models as follows: (iv) using mean healthy range values for vital sign and lab test information for days prior to the first measurement and LOCF thereafter but excluding respiratory rate, PO2A, and PHA (stabilized through ventilation), (v) using mean healthy range values for vital sign and lab test information for days prior to the first measurement and LOCF thereafter but excluding pulse and systolic blood pressure (stabilized by vasoactive infusion), and (vi) using mean healthy range values for vital sign and lab test information for days prior to the first measurement and LOCF thereafter but excluding sodium, potassium, creatinine, blood urea nitrogen, and bicarbonate (stabilized by CRRT).

These seven Cox models (1 for the primary analysis and 6 for sensitivity analyses) were estimated for both study outcomes; any DNR and comfort care. **Supplementary Figures b and c** summarize the results of these models. All models were similar in results. The exception being the model excluding the vital signs and Lab values, where the use of mechanical ventilation was significantly associated with change to Comfort Care or Any DNR**.**

Supplementary Table a: Frequency and percent of Non-missing values for vital signs and lab tests

| Vital signs and Lab values | Day 1 | Day 2 | Day 3 | Day 4 |
| --- | --- | --- | --- | --- |
| Blood Urea Nitrogen (BUN) (mg/dl) | 4492 (44%) | 9623 (95%) | 9737 (99%) | 9262 (95%) |
| Bicarbonate (mEq/L) | 4528 (45%) | 9669 (96%) | 9767 (100%) | 9280 (95%) |
| Creatinine (CR) (mg/dl) | 4587 (45%) | 9697 (96%) | 9771 (100%) | 9282 (95%) |
| Glasgow Coma Score (GCS) | 890 (9%) | 3542 (35%) | 3781 (39%) | 3446 (35%) |
| Hematocrit (HCT) (%) | 4712 (46%) | 9878 (98%) | 9778 (100%) | 9285 (95%) |
| Arterial PH (pHa) | 1567 (15%) | 8280 (82%) | 8588 (88%) | 8301 (85%) |
| Partial oxygen pressure (pO2a) | 1669 (16%) | 8398 (83%) | 8660 (88%) | 8365 (85%) |
| Potassium (mEq/L) | 4716 (46%) | 9880 (98%) | 9779 (100%) | 9287 (95%) |
| Pulse (per 10 bpm) | 4914 (48%) | 9995 (99%) | 9612 (98%) | 9135 (93%) |
| Respiratory Rate (breaths/min) | 4894 (48%) | 9988 (99%) | 9619 (98%) | 9135 (93%) |
| Systolic Blood Pressure (mmHg) (per 10mmHg) | 4827 (48%) | 9539 (94%) | 7795 (80%) | 7605 (78%) |
| Sodium (mEq/L) | 4714 (46%) | 9875 (98%) | 9779 (100%) | 9287 (95%) |
| Total bilirubin (mg/dl) | 2788 (27%) | 6619 (66%) | 7331 (75%) | 7225 (74%) |
| Temperature (F) | 4808 (47%) | 10015 (99%) | 9543 (97%) | 9006 (92%) |
| White blood cells (10^9/L) | 4556 (45%) | 9698 (96%) | 9754 (100%) | 9272 (95%) |

Supplementary Table b: Frequency and percent of Non-missing values for vital signs and lab tests after missing values were replaced using last value carried forward

| Vital signs and Lab values | Day 1 | Day 2 | Day 3 | Day 4 |
| --- | --- | --- | --- | --- |
| Blood Urea Nitrogen (BUN) (mg/dl) | 4492 (44%) | 9626 (95%) | 9268 (100%) | 8701 (100%) |
| Bicarbonate (mEq/L) | 4528 (45%) | 9671 (96%) | 9282 (100%) | 8709 (100%) |
| Creatinine (CR) (mg/dl) | 4587 (45%) | 9699 (96%) | 9284 (100%) | 8711 (100%) |
| Glasgow Coma Score (GCS) | 890 (9%) | 3558 (35%) | 3766 (41%) | 3600 (41%) |
| Hematocrit (HCT) (%) | 4712 (46%) | 9879 (98%) | 9286 (100%) | 8712 (100%) |
| Arterial PH (pHa) | 1567 (15%) | 8284 (82%) | 8316 (89%) | 7909 (91%) |
| Partial oxygen pressure (pO2a) | 1669 (16%) | 8402 (83%) | 8381 (90%) | 7964 (91%) |
| Potassium (mEq/L) | 4716 (46%) | 9881 (98%) | 9288 (100%) | 8712 (100%) |
| Pulse (per 10 bpm) | 4914 (48%) | 10048 (100%) | 9283 (100%) | 8706 (100%) |
| Respiratory Rate (breaths/min) | 4894 (48%) | 10037 (99%) | 9285 (100%) | 8707 (100%) |
| Systolic Blood Pressure (mmHg) (per 10mmHg) | 4827 (48%) | 9807 (97%) | 9242 (99%) | 8684 (100%) |
| Sodium (mEq/L) | 4714 (46%) | 9876 (98%) | 9288 (100%) | 8712 (100%) |
| Total billirubin (mg/dl) | 2788 (27%) | 6633 (66%) | 7257 (78%) | 6960 (80%) |
| Temperature (F) | 4808 (47%) | 10030 (99%) | 9286 (100%) | 8706 (100%) |
| White blood cells (10^9/L) | 4556 (45%) | 9700 (96%) | 9273 (100%) | 8707 (100%) |

**Supplementary Table c**: Vital sign and lab test healthy ranges and correspondence with medical support

| **Vital sign or lab test** | **Healthy range values** | **Stabilized through medical support** | | |
| --- | --- | --- | --- | --- |
|  | **mean (min, max)** | **Vasoactive**  **infusion** | **Ventilation** | **CRRT** |
| Temperature (F) | 98.6 (96.8, 101) |  |  |  |
| Systolic Blood Pressure (mmHg) | 110 (100,199) | Yes |  |  |
| Pulse (Beats/min) | 80 (70, 109) | Yes |  |  |
| Respiratory rate (Breaths/min) | 16 (12, 24) |  | Yes |  |
| PO2A (mmHg) | 95 (70, 100) |  | Yes |  |
| PHA | 7.4 (7.33, 7.49) |  | Yes |  |
| Sodium (mEq/L) | 145 (130, 149) |  |  | Yes |
| Potassium (mEq/L) | 4.5 (3.5, 5.4) |  |  | Yes |
| Creatinine (mg/dl) | 0.8 (0.6, 1.4) |  |  | Yes |
| Hematocrit (%) | 42 (30, 45.9) |  |  |  |
| White blood cells (count × 10^9^/L) | 8 (3, 14.9) |  |  |  |
| Glasgow Coma Scale score | 15 (15, 15) |  |  |  |
| Blood urea nitrogen (mg/dl) | 15 (n/a, 28) |  |  | Yes |
| Total bilirubin (mg/dl) | 1 (n/a, 4) |  |  | Yes |
| Bicarbonate (mEq/L) | 24 (20, n/a) |  |  |  |

**Supplementary Figure a:** Example pseudo-record

| Pat. ID | Day | SBP | GCS | Pulse | TBIL | Vasoactive | CRRT | Ventilator | Charlson.COPD | Charlson.  Dementia | CV-ICU | MT-Med | MI-ICU | CC |
| --- | --- | --- | --- | --- | --- | --- | --- | --- | --- | --- | --- | --- | --- | --- |
| X001 | 1 | 98 |  | 92 |  | 1 | 0 | 0 | 0 | 1 | 0 | 1 | 0 | 0 |
| X001 | 2 | 53 |  | 85 | 0.5 | 1 | 0 | 1 | 0 | 1 | 0 | 1 | 0 | 0 |
| X001 | 3 | 97 | 13 | 106 |  | 0 | 0 | 1 | 0 | 1 | 0 | 1 | 0 | 0 |
| X001 | 4 | 110 | 9 | 57 | 0.7 | 1 | 0 | 1 | 0 | 1 | 0 | 1 | 1 | 0 |
| X001 | 5 | 92 | 9 | 74 |  | 0 | 0 | 1 | 0 | 1 | 0 | 1 | 1 | 0 |
| X001 | 6 | 120 | 8 | 85 | 0.7 | 1 | 0 | 1 | 0 | 1 | 0 | 1 | 1 | 0 |
| X001 | 7 | 101 | 11 | 89 | 0.7 | 1 | 0 | 1 | 0 | 1 | 0 | 1 | 1 | 1 |

**SBP=Systolic Blood Pressure, GCS=Glasgow Coma Score, TBIL=Total bilirubin, Charlson. COPD= Charlson Chronic Obstructive Pulmonary Disorder, CV-ICU= Cardiovascular Intensive Care Unit, MT-Med= Medical Team Medicine, MI-ICU= Myocardial Infarction Intensive Care Unit , CC= Comfort Care initiated**

**Supplementary Figure b:** Comparison of coefficients from primary and sensitivity analyses for comfort care
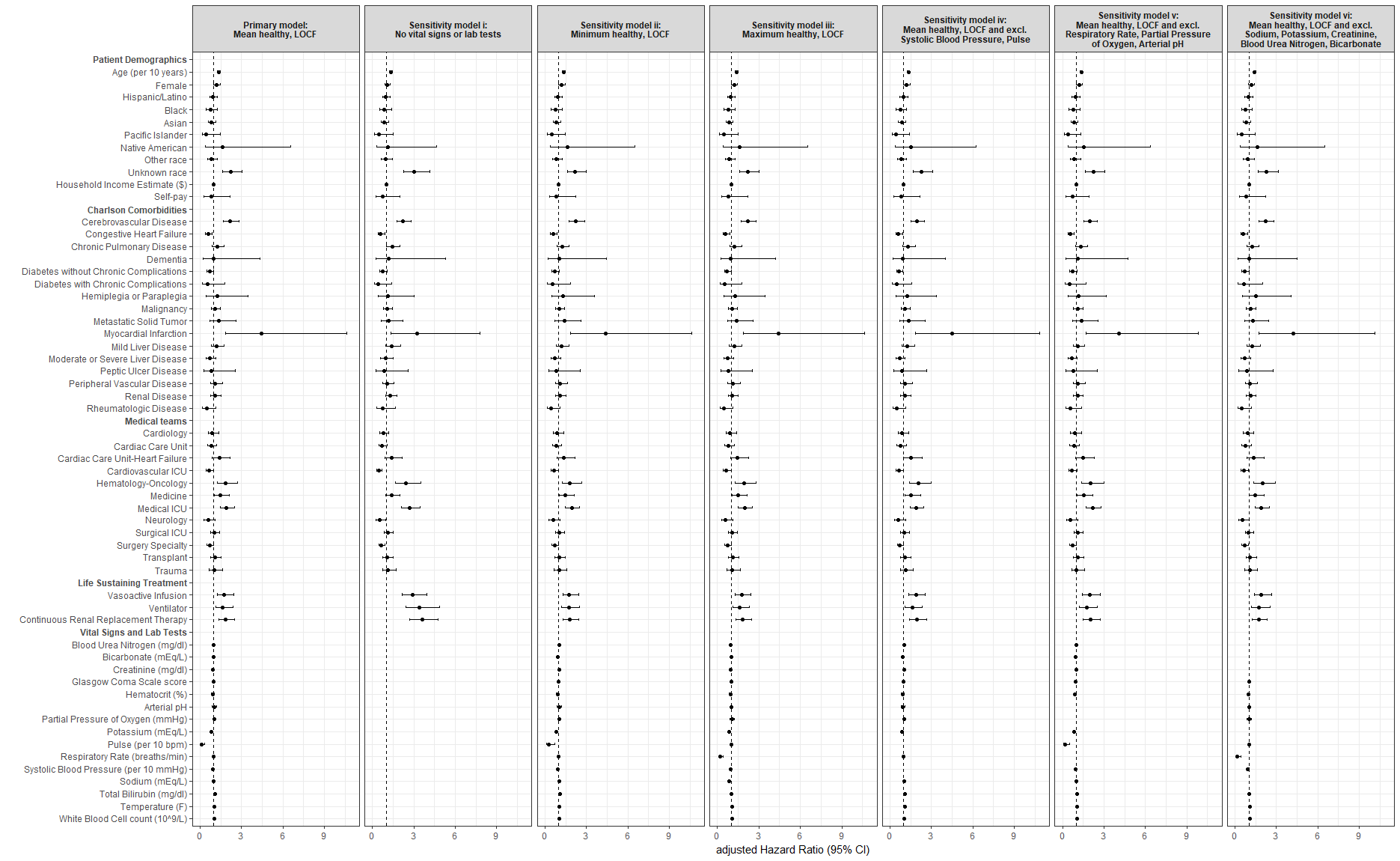


LOCF = last observation carried forward, ICU = Intensive care unit.

**Supplementary Figure c:** Comparison of coefficients from primary and sensitivity analyses for Any DNR
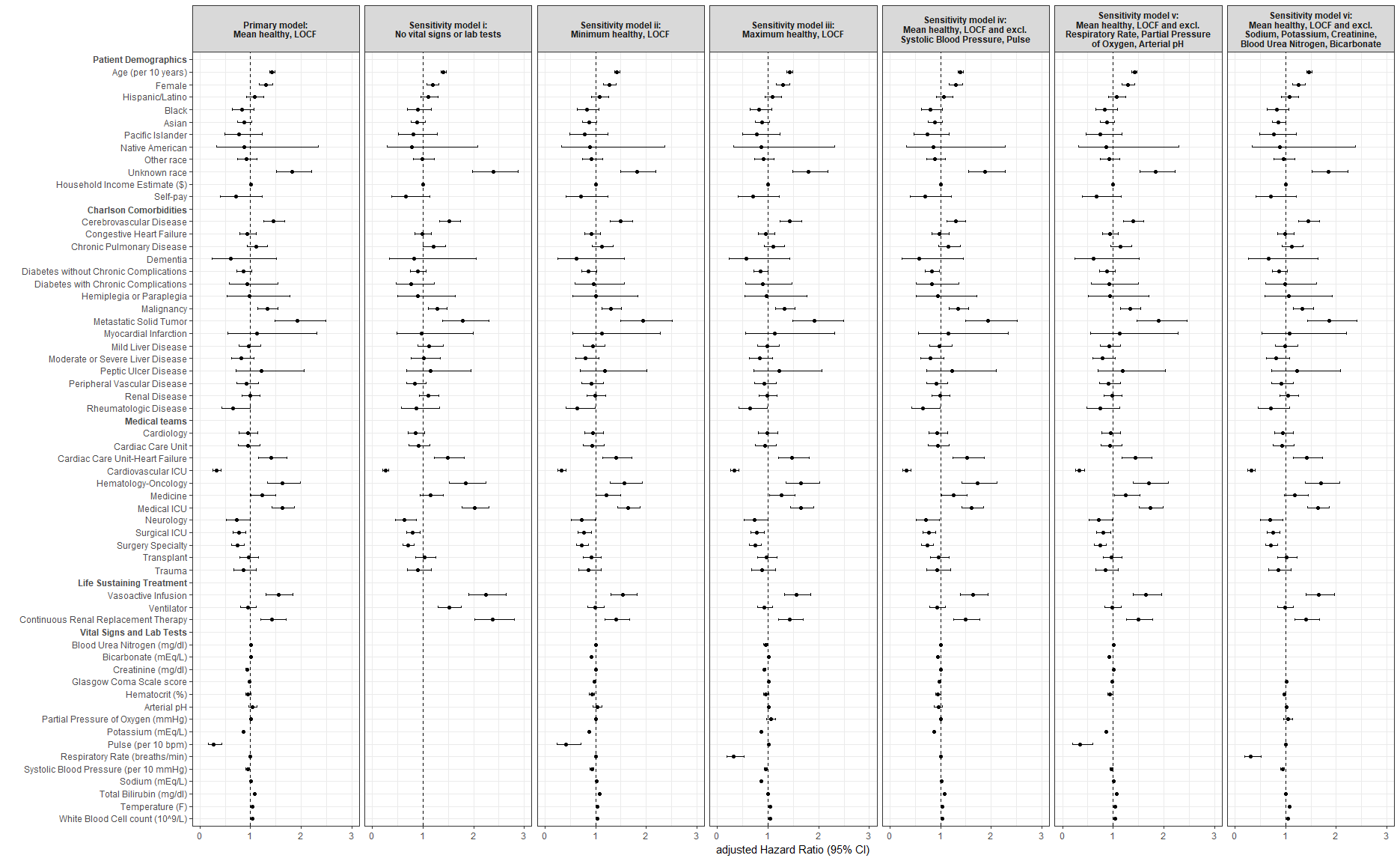


LOCF = last observation carried forward, ICU = Intensive care unit.

**Supplementary Table c:** Comparison of coefficients from primary and sensitivity analyses for Comfort Care.

| Variables | Main Model | i. Fixed Model | ii. Minimum Model | iii. Maximum Model | iv Ventillator Model | v Vasoactive Model | vi. CRRT Model |
| --- | --- | --- | --- | --- | --- | --- | --- |
| Patient Demographics |  |  |  |  |  |  |  |
| Age (per 10 years) | 1.37 [95% CI 1.28-1.47] | 1.34 [95% CI 1.25-1.43] | 1.36 [95% CI 1.28-1.46] | 1.37 [95% CI 1.28-1.47] | 1.36 [95% CI 1.27-1.46] | 1.34 [95% CI 1.25-1.43] | 1.42 [95% CI 1.32-1.51] |
| Female | 1.21 [95% CI 0.99-1.47] | 1.08 [95% CI 0.89-1.3] | 1.19 [95% CI 0.98-1.44] | 1.2 [95% CI 0.99-1.46] | 1.2 [95% CI 0.99-1.46] | 1.2 [95% CI 0.99-1.46] | 1.17 [95% CI 0.96-1.41] |
| Hispanic/Latino | 0.95 [95% CI 0.71-1.28] | 0.96 [95% CI 0.72-1.29] | 0.95 [95% CI 0.7-1.27] | 0.96 [95% CI 0.71-1.29] | 0.92 [95% CI 0.69-1.24] | 0.95 [95% CI 0.71-1.28] | 0.95 [95% CI 0.7-1.27] |
| African-American | 0.77 [95% CI 0.47-1.25] | 0.85 [95% CI 0.52-1.38] | 0.76 [95% CI 0.46-1.24] | 0.77 [95% CI 0.47-1.26] | 0.79 [95% CI 0.48-1.29] | 0.74 [95% CI 0.45-1.22] | 0.76 [95% CI 0.46-1.23] |
| Asian | 0.83 [95% CI 0.61-1.13] | 0.87 [95% CI 0.64-1.18] | 0.82 [95% CI 0.6-1.12] | 0.83 [95% CI 0.61-1.14] | 0.83 [95% CI 0.61-1.13] | 0.84 [95% CI 0.62-1.15] | 0.81 [95% CI 0.59-1.11] |
| Pacific islander | 0.47 [95% CI 0.15-1.48] | 0.47 [95% CI 0.15-1.47] | 0.47 [95% CI 0.15-1.49] | 0.47 [95% CI 0.15-1.49] | 0.42 [95% CI 0.13-1.33] | 0.45 [95% CI 0.14-1.4] | 0.46 [95% CI 0.14-1.44] |
| Native American | 1.62 [95% CI 0.4-6.55] | 1.14 [95% CI 0.28-4.61] | 1.61 [95% CI 0.4-6.52] | 1.62 [95% CI 0.4-6.54] | 1.56 [95% CI 0.39-6.34] | 1.54 [95% CI 0.38-6.24] | 1.61 [95% CI 0.4-6.51] |
| Other | 0.83 [95% CI 0.55-1.27] | 0.96 [95% CI 0.63-1.45] | 0.84 [95% CI 0.55-1.28] | 0.83 [95% CI 0.55-1.27] | 0.86 [95% CI 0.56-1.3] | 0.8 [95% CI 0.52-1.22] | 0.92 [95% CI 0.61-1.4] |
| Unknown | 2.21 [95% CI 1.61-3.02] | 3.04 [95% CI 2.23-4.15] | 2.19 [95% CI 1.6-3] | 2.2 [95% CI 1.61-3.02] | 2.24 [95% CI 1.64-3.07] | 2.27 [95% CI 1.66-3.1] | 2.27 [95% CI 1.66-3.11] |
| Household Income Estimate ($) | 1 [95% CI 1-1] | 1 [95% CI 1-1] | 1 [95% CI 1-1] | 1 [95% CI 1-1] | 1 [95% CI 1-1] | 1 [95% CI 1-1] | 1 [95% CI 1-1] |
| Self-Pay | 0.82 [95% CI 0.3-2.2] | 0.73 [95% CI 0.27-1.96] | 0.82 [95% CI 0.31-2.23] | 0.81 [95% CI 0.3-2.17] | 0.72 [95% CI 0.27-1.94] | 0.8 [95% CI 0.3-2.16] | 0.82 [95% CI 0.3-2.21] |
| Charlson Comorbidities |  |  |  |  |  |  |  |
| Cerebrovascular Disease | 2.18 [95% CI 1.69-2.81] | 2.22 [95% CI 1.75-2.82] | 2.25 [95% CI 1.74-2.9] | 2.18 [95% CI 1.69-2.8] | 1.97 [95% CI 1.53-2.54] | 1.95 [95% CI 1.51-2.5] | 2.2 [95% CI 1.71-2.82] |
| Congestive Heart Failure | 0.59 [95% CI 0.4-0.86] | 0.6 [95% CI 0.41-0.88] | 0.58 [95% CI 0.4-0.85] | 0.6 [95% CI 0.41-0.87] | 0.57 [95% CI 0.39-0.84] | 0.62 [95% CI 0.42-0.9] | 0.61 [95% CI 0.42-0.89] |
| COPD | 1.25 [95% CI 0.89-1.76] | 1.43 [95% CI 1.02-1.99] | 1.25 [95% CI 0.89-1.76] | 1.24 [95% CI 0.88-1.74] | 1.3 [95% CI 0.92-1.83] | 1.29 [95% CI 0.92-1.82] | 1.25 [95% CI 0.89-1.75] |
| Dementia | 1 [95% CI 0.23-4.35] | 1.19 [95% CI 0.27-5.27] | 1.03 [95% CI 0.24-4.47] | 0.96 [95% CI 0.22-4.21] | 1.09 [95% CI 0.25-4.76] | 0.91 [95% CI 0.21-4] | 1.02 [95% CI 0.23-4.49] |
| Diabetes without Chronic Complications | 0.71 [95% CI 0.5-1.01] | 0.75 [95% CI 0.53-1.06] | 0.72 [95% CI 0.51-1.01] | 0.7 [95% CI 0.5-1] | 0.74 [95% CI 0.52-1.04] | 0.67 [95% CI 0.47-0.94] | 0.71 [95% CI 0.5-1] |
| Diabetes with Chronic Complications | 0.56 [95% CI 0.17-1.82] | 0.44 [95% CI 0.14-1.41] | 0.57 [95% CI 0.17-1.84] | 0.54 [95% CI 0.17-1.76] | 0.53 [95% CI 0.16-1.73] | 0.48 [95% CI 0.15-1.57] | 0.62 [95% CI 0.19-2.01] |
| Hemiplagia or Paraplegia | 1.27 [95% CI 0.47-3.48] | 1.12 [95% CI 0.41-3.01] | 1.31 [95% CI 0.48-3.58] | 1.26 [95% CI 0.46-3.43] | 1.16 [95% CI 0.42-3.18] | 1.23 [95% CI 0.45-3.34] | 1.5 [95% CI 0.55-4.04] |
| Malignancy | 1.08 [95% CI 0.8-1.45] | 1.07 [95% CI 0.8-1.43] | 1.06 [95% CI 0.79-1.42] | 1.07 [95% CI 0.8-1.45] | 1.09 [95% CI 0.81-1.47] | 1.1 [95% CI 0.82-1.48] | 1.11 [95% CI 0.83-1.5] |
| Metastatic Solid Tumor | 1.37 [95% CI 0.72-2.6] | 1.17 [95% CI 0.62-2.2] | 1.39 [95% CI 0.73-2.63] | 1.36 [95% CI 0.71-2.58] | 1.36 [95% CI 0.72-2.58] | 1.35 [95% CI 0.71-2.57] | 1.29 [95% CI 0.68-2.44] |
| Myocardial Infarction | 4.43 [95% CI 1.85-10.62 | 3.23 [95% CI 1.34-7.76] | 4.41 [95% CI 1.83-10.62 | 4.44 [95% CI 1.85-10.67 | 4.09 [95% CI 1.69-9.86] | 4.5 [95% CI 1.87-10.85] | 4.2 [95% CI 1.74-10.13] |
| Mild Liver Disease | 1.19 [95% CI 0.82-1.73] | 1.42 [95% CI 0.97-2.06] | 1.18 [95% CI 0.82-1.72] | 1.21 [95% CI 0.84-1.76] | 1.11 [95% CI 0.76-1.62] | 1.24 [95% CI 0.86-1.79] | 1.24 [95% CI 0.85-1.82] |
| Moderate or Severe Liver Disease | 0.72 [95% CI 0.45-1.14] | 0.93 [95% CI 0.58-1.49] | 0.71 [95% CI 0.45-1.12] | 0.73 [95% CI 0.46-1.16] | 0.65 [95% CI 0.41-1.04] | 0.7 [95% CI 0.44-1.1] | 0.72 [95% CI 0.45-1.15] |
| Peptic Ulcer | 0.81 [95% CI 0.26-2.58] | 0.82 [95% CI 0.26-2.58] | 0.8 [95% CI 0.25-2.53] | 0.8 [95% CI 0.25-2.54] | 0.79 [95% CI 0.25-2.51] | 0.84 [95% CI 0.27-2.67] | 0.87 [95% CI 0.27-2.75] |
| Peripheral Vascular Disease | 1.1 [95% CI 0.75-1.63] | 1.08 [95% CI 0.74-1.57] | 1.1 [95% CI 0.75-1.63] | 1.1 [95% CI 0.75-1.63] | 1.12 [95% CI 0.76-1.66] | 1.11 [95% CI 0.75-1.64] | 1.09 [95% CI 0.74-1.61] |
| Renal Disease | 1.08 [95% CI 0.77-1.51] | 1.28 [95% CI 0.93-1.76] | 1.1 [95% CI 0.79-1.53] | 1.07 [95% CI 0.77-1.49] | 1.09 [95% CI 0.78-1.51] | 1.08 [95% CI 0.78-1.51] | 1.11 [95% CI 0.81-1.54] |
| Rheumatologic Disease | 0.47 [95% CI 0.2-1.13] | 0.71 [95% CI 0.31-1.66] | 0.45 [95% CI 0.19-1.09] | 0.47 [95% CI 0.2-1.11] | 0.57 [95% CI 0.24-1.37] | 0.47 [95% CI 0.2-1.12] | 0.49 [95% CI 0.21-1.17] |
| Medical Teams |  |  |  |  |  |  |  |
| Cardiology | 0.89 [95% CI 0.59-1.35] | 0.77 [95% CI 0.52-1.16] | 0.89 [95% CI 0.58-1.35] | 0.92 [95% CI 0.6-1.4] | 0.89 [95% CI 0.59-1.36] | 0.89 [95% CI 0.58-1.35] | 0.89 [95% CI 0.59-1.35] |
| Cardiac Care Unit (CCU) | 0.8 [95% CI 0.53-1.22] | 0.7 [95% CI 0.46-1.07] | 0.79 [95% CI 0.52-1.21] | 0.8 [95% CI 0.52-1.22] | 0.81 [95% CI 0.53-1.24] | 0.78 [95% CI 0.51-1.2] | 0.77 [95% CI 0.5-1.17] |
| Cardiac Care Unit-Heart Failure (CCU(HF)) | 1.4 [95% CI 0.9-2.18] | 1.38 [95% CI 0.89-2.14] | 1.38 [95% CI 0.89-2.16] | 1.45 [95% CI 0.93-2.26] | 1.48 [95% CI 0.95-2.3] | 1.51 [95% CI 0.97-2.35] | 1.36 [95% CI 0.87-2.12] |
| Cardiovascular ICU (CVICU) | 0.64 [95% CI 0.42-0.98] | 0.46 [95% CI 0.31-0.68] | 0.64 [95% CI 0.42-0.98] | 0.65 [95% CI 0.42-1] | 0.69 [95% CI 0.45-1.05] | 0.64 [95% CI 0.42-0.98] | 0.63 [95% CI 0.41-0.95] |
| Hematology-Oncology | 1.87 [95% CI 1.27-2.74] | 2.42 [95% CI 1.66-3.53] | 1.8 [95% CI 1.23-2.63] | 1.9 [95% CI 1.3-2.79] | 2.04 [95% CI 1.39-2.99] | 2.03 [95% CI 1.39-2.98] | 2 [95% CI 1.36-2.94] |
| Medicine | 1.48 [95% CI 1.02-2.13] | 1.38 [95% CI 0.96-1.99] | 1.48 [95% CI 1.02-2.13] | 1.5 [95% CI 1.04-2.16] | 1.52 [95% CI 1.06-2.19] | 1.54 [95% CI 1.07-2.23] | 1.45 [95% CI 1.01-2.1] |
| Medical ICU | 1.92 [95% CI 1.49-2.49] | 2.67 [95% CI 2.08-3.43] | 1.93 [95% CI 1.49-2.5] | 1.96 [95% CI 1.51-2.54] | 2.17 [95% CI 1.68-2.8] | 1.9 [95% CI 1.47-2.46] | 1.92 [95% CI 1.48-2.47] |
| Neurology | 0.58 [95% CI 0.3-1.12] | 0.5 [95% CI 0.26-0.96] | 0.58 [95% CI 0.3-1.11] | 0.58 [95% CI 0.3-1.12] | 0.59 [95% CI 0.31-1.13] | 0.58 [95% CI 0.3-1.12] | 0.54 [95% CI 0.28-1.04] |
| Surgical ICU | 1.03 [95% CI 0.76-1.4] | 1.13 [95% CI 0.85-1.52] | 1.04 [95% CI 0.77-1.41] | 1.04 [95% CI 0.77-1.42] | 1.11 [95% CI 0.82-1.5] | 1.03 [95% CI 0.76-1.39] | 0.99 [95% CI 0.73-1.34] |
| Surgery Specialty | 0.72 [95% CI 0.52-1] | 0.65 [95% CI 0.47-0.89] | 0.71 [95% CI 0.51-0.98] | 0.72 [95% CI 0.52-0.99] | 0.71 [95% CI 0.52-0.98] | 0.71 [95% CI 0.51-0.99] | 0.69 [95% CI 0.5-0.95] |
| Transplant | 1.08 [95% CI 0.77-1.52] | 1.07 [95% CI 0.75-1.51] | 1.03 [95% CI 0.73-1.44] | 1.09 [95% CI 0.78-1.54] | 1.1 [95% CI 0.78-1.55] | 1.08 [95% CI 0.77-1.51] | 1.1 [95% CI 0.79-1.55] |
| Trauma | 1.05 [95% CI 0.68-1.61] | 1.11 [95% CI 0.72-1.71] | 1.03 [95% CI 0.67-1.58] | 1.06 [95% CI 0.69-1.63] | 1.02 [95% CI 0.66-1.58] | 1.12 [95% CI 0.73-1.7] | 1.05 [95% CI 0.68-1.63] |
| Life Sustaining Treatment |  |  |  |  |  |  |  |
| Vasoactive | 1.76 [95% CI 1.28-2.43] | 2.88 [95% CI 2.13-3.91] | 1.76 [95% CI 1.28-2.42] | 1.76 [95% CI 1.28-2.43] | 1.98 [95% CI 1.44-2.72] | 1.87 [95% CI 1.36-2.57] | 1.91 [95% CI 1.39-2.63] |
| Ventilator | 1.63 [95% CI 1.12-2.38] | 3.42 [95% CI 2.41-4.84] | 1.72 [95% CI 1.18-2.52] | 1.59 [95% CI 1.09-2.31] | 1.74 [95% CI 1.2-2.54] | 1.62 [95% CI 1.11-2.35] | 1.74 [95% CI 1.19-2.53] |
| CCRT | 1.83 [95% CI 1.34-2.48] | 3.59 [95% CI 2.7-4.77] | 1.8 [95% CI 1.33-2.44] | 1.83 [95% CI 1.35-2.49] | 2.02 [95% CI 1.49-2.73] | 1.94 [95% CI 1.43-2.63] | 1.71 [95% CI 1.26-2.32] |
| Vital Signs and Lab Tests |  |  |  |  |  |  |  |
| Blood Urea Nitrogen (BUN) (mg/dl) | 1.01 [95% CI 1.01-1.02] | NA | 1.02 [95% CI 1.01-1.02] | 1.01 [95% CI 1.01-1.02] | 1.02 [95% CI 1.01-1.02] | 1.01 [95% CI 1.01-1.02] | NA |
| Bicarbonate (mEq/L) | 0.98 [95% CI 0.95-1] | NA | 0.97 [95% CI 0.95-0.99] | 0.98 [95% CI 0.95-1] | 0.96 [95% CI 0.94-0.98] | 0.97 [95% CI 0.95-0.99] | NA |
| Creatinine (CR) (mg/dl) | 0.92 [95% CI 0.83-1.02] | NA | 0.9 [95% CI 0.82-1] | 0.92 [95% CI 0.83-1.03] | 0.91 [95% CI 0.82-1.01] | 0.92 [95% CI 0.83-1.02] | NA |
| Glasgow Coma Score (GCS) | 0.84 [95% CI 0.82-0.86] | NA | 0.84 [95% CI 0.82-0.86] | 0.84 [95% CI 0.82-0.86] | 0.83 [95% CI 0.81-0.85] | 0.84 [95% CI 0.82-0.86] | 0.83 [95% CI 0.81-0.86] |
| Hematocrit (HCT) (%) | 1.01 [95% CI 1-1.03] | NA | 1 [95% CI 0.98-1.02] | 1.01 [95% CI 1-1.03] | 1.01 [95% CI 0.99-1.03] | 1.01 [95% CI 0.99-1.03] | 1.01 [95% CI 0.99-1.02] |
| Arterial PH (pHa) | 0.2 [95% CI 0.07-0.53] | NA | 0.17 [95% CI 0.07-0.43] | 0.25 [95% CI 0.09-0.7] | NA | 0.17 [95% CI 0.07-0.42] | 0.13 [95% CI 0.06-0.32] |
| Partial oxygen pressure (pO2a) | 1 [95% CI 1-1] | NA | 1 [95% CI 1-1] | 1 [95% CI 1-1] | NA | 1 [95% CI 1-1] | 1 [95% CI 1-1] |
| Potassium (mEq/L) | 1.01 [95% CI 0.86-1.18] | NA | 0.93 [95% CI 0.8-1.09] | 1.02 [95% CI 0.88-1.19] | 1.05 [95% CI 0.9-1.23] | 1.01 [95% CI 0.87-1.18] | NA |
| Pulse (per 10 bpm) | 1.07 [95% CI 1.05-1.09] | NA | 1.07 [95% CI 1.05-1.09] | 1.07 [95% CI 1.05-1.09] | 1.08 [95% CI 1.06-1.1] | NA | 1.07 [95% CI 1.05-1.09] |
| Respiratory Rate (breaths/min) | 1.06 [95% CI 1.05-1.07] | NA | 1.06 [95% CI 1.04-1.07] | 1.06 [95% CI 1.05-1.08] | NA | 1.06 [95% CI 1.05-1.08] | 1.06 [95% CI 1.05-1.08] |
| Systolic Blood Pressure (per 10mmHg) | 0.92 [95% CI 0.88-0.96] | NA | 0.92 [95% CI 0.88-0.96] | 0.92 [95% CI 0.89-0.96] | 0.92 [95% CI 0.88-0.96] | NA | 0.91 [95% CI 0.87-0.95] |
| Sodium (mEq/L) | 1.02 [95% CI 1-1.03] | NA | 1.01 [95% CI 0.99-1.02] | 1.02 [95% CI 1-1.03] | 1.02 [95% CI 1-1.03] | 1.02 [95% CI 1-1.03] | NA |
| Total bilirubin (mg/dl) | 1.05 [95% CI 1.03-1.06] | NA | 1.04 [95% CI 1.03-1.06] | 1.04 [95% CI 1.03-1.06] | 1.04 [95% CI 1.03-1.06] | 1.04 [95% CI 1.03-1.06] | 1.05 [95% CI 1.04-1.06] |
| Temperature (F) | 0.95 [95% CI 0.89-1] | NA | 0.93 [95% CI 0.88-0.99] | 0.95 [95% CI 0.9-1.01] | 0.95 [95% CI 0.9-1.02] | 0.96 [95% CI 0.91-1.02] | 0.94 [95% CI 0.88-1] |
| White blood cells (10^9/L) | 1.01 [95% CI 1-1.01] | NA | 1.01 [95% CI 1-1.01] | 1.01 [95% CI 1-1.01] | 1.01 [95% CI 1-1.01] | 1.01 [95% CI 1-1.01] | 1.01 [95% CI 1-1.01] |

**Supplementary Table d:** Comparison of coefficients from primary and sensitivity analyses for Any DNR

| Variables | Main Model | i. Fixed Model | ii. Minimum Model | iii. Maximum Model | iv Ventillator Model | v Vasoactive Model | vi. CRRT Model |
| --- | --- | --- | --- | --- | --- | --- | --- |
| Patient Demographics |  |  |  |  |  |  |  |
| Age (per 10 years) | 1.43 [95% CI 1.38-1.48] | 1.4 [95% CI 1.35-1.45] | 1.42 [95% CI 1.37-1.48] | 1.43 [95% CI 1.38-1.48] | 1.42 [95% CI 1.37-1.48] | 1.39 [95% CI 1.34-1.44] | 1.46 [95% CI 1.41-1.52] |
| Female | 1.3 [95% CI 1.17-1.44] | 1.19 [95% CI 1.08-1.31] | 1.28 [95% CI 1.15-1.41] | 1.29 [95% CI 1.17-1.43] | 1.29 [95% CI 1.17-1.43] | 1.3 [95% CI 1.17-1.44] | 1.26 [95% CI 1.14-1.39] |
| Hispanic/Latino | 1.08 [95% CI 0.92-1.26] | 1.11 [95% CI 0.95-1.29] | 1.08 [95% CI 0.92-1.26] | 1.09 [95% CI 0.93-1.27] | 1.07 [95% CI 0.91-1.24] | 1.07 [95% CI 0.92-1.25] | 1.07 [95% CI 0.92-1.25] |
| African-American | 0.83 [95% CI 0.64-1.07] | 0.9 [95% CI 0.69-1.16] | 0.83 [95% CI 0.64-1.07] | 0.82 [95% CI 0.64-1.07] | 0.84 [95% CI 0.65-1.09] | 0.8 [95% CI 0.62-1.04] | 0.82 [95% CI 0.64-1.06] |
| Asian | 0.87 [95% CI 0.75-1.02] | 0.89 [95% CI 0.76-1.04] | 0.87 [95% CI 0.74-1.02] | 0.88 [95% CI 0.75-1.03] | 0.87 [95% CI 0.74-1.02] | 0.88 [95% CI 0.75-1.04] | 0.86 [95% CI 0.73-1] |
| Pacific islander | 0.78 [95% CI 0.49-1.23] | 0.81 [95% CI 0.51-1.28] | 0.78 [95% CI 0.49-1.24] | 0.78 [95% CI 0.49-1.23] | 0.74 [95% CI 0.47-1.18] | 0.74 [95% CI 0.47-1.17] | 0.76 [95% CI 0.48-1.2] |
| Native American | 0.87 [95% CI 0.33-2.34] | 0.78 [95% CI 0.29-2.08] | 0.88 [95% CI 0.33-2.36] | 0.87 [95% CI 0.32-2.32] | 0.86 [95% CI 0.32-2.3] | 0.85 [95% CI 0.32-2.28] | 0.89 [95% CI 0.33-2.38] |
| Other | 0.91 [95% CI 0.74-1.12] | 0.99 [95% CI 0.8-1.22] | 0.92 [95% CI 0.75-1.14] | 0.91 [95% CI 0.74-1.12] | 0.92 [95% CI 0.75-1.13] | 0.89 [95% CI 0.72-1.09] | 0.95 [95% CI 0.77-1.17] |
| Unknown | 1.82 [95% CI 1.5-2.2] | 2.38 [95% CI 1.98-2.88] | 1.81 [95% CI 1.5-2.19] | 1.8 [95% CI 1.49-2.18] | 1.84 [95% CI 1.52-2.23] | 1.88 [95% CI 1.56-2.27] | 1.84 [95% CI 1.52-2.23] |
| Household Income Estimate ($) | 1 [95% CI 1-1] | 1 [95% CI 1-1] | 1 [95% CI 1-1] | 1 [95% CI 1-1] | 1 [95% CI 1-1] | 1 [95% CI 1-1] | 1 [95% CI 1-1] |
| Self-Pay | 0.71 [95% CI 0.41-1.22] | 0.66 [95% CI 0.38-1.14] | 0.71 [95% CI 0.41-1.24] | 0.7 [95% CI 0.41-1.22] | 0.67 [95% CI 0.39-1.16] | 0.7 [95% CI 0.4-1.21] | 0.7 [95% CI 0.41-1.21] |
| Charlson Comorbidities |  |  |  |  |  |  |  |
| Cerebrovascular Disease | 1.45 [95% CI 1.25-1.67] | 1.52 [95% CI 1.32-1.74] | 1.5 [95% CI 1.29-1.73] | 1.43 [95% CI 1.24-1.66] | 1.39 [95% CI 1.2-1.61] | 1.3 [95% CI 1.12-1.5] | 1.45 [95% CI 1.25-1.67] |
| Congestive Heart Failure | 0.94 [95% CI 0.79-1.12] | 0.99 [95% CI 0.83-1.17] | 0.92 [95% CI 0.78-1.1] | 0.96 [95% CI 0.8-1.13] | 0.93 [95% CI 0.79-1.11] | 0.98 [95% CI 0.83-1.16] | 0.99 [95% CI 0.84-1.17] |
| COPD | 1.11 [95% CI 0.93-1.34] | 1.2 [95% CI 1-1.44] | 1.12 [95% CI 0.93-1.35] | 1.11 [95% CI 0.92-1.33] | 1.14 [95% CI 0.95-1.37] | 1.15 [95% CI 0.96-1.39] | 1.12 [95% CI 0.94-1.35] |
| Dementia | 0.6 [95% CI 0.24-1.5] | 0.83 [95% CI 0.33-2.06] | 0.63 [95% CI 0.25-1.56] | 0.57 [95% CI 0.23-1.43] | 0.61 [95% CI 0.24-1.52] | 0.58 [95% CI 0.23-1.45] | 0.66 [95% CI 0.27-1.64] |
| Diabetes without Chronic Complications | 0.86 [95% CI 0.73-1.02] | 0.89 [95% CI 0.75-1.05] | 0.87 [95% CI 0.73-1.03] | 0.85 [95% CI 0.72-1] | 0.87 [95% CI 0.74-1.04] | 0.82 [95% CI 0.7-0.98] | 0.87 [95% CI 0.73-1.03] |
| Diabetes with Chronic Complications | 0.94 [95% CI 0.58-1.53] | 0.76 [95% CI 0.47-1.23] | 0.96 [95% CI 0.59-1.56] | 0.9 [95% CI 0.55-1.47] | 0.92 [95% CI 0.56-1.51] | 0.83 [95% CI 0.51-1.36] | 0.99 [95% CI 0.61-1.61] |
| Hemiplagia or Paraplegia | 0.98 [95% CI 0.54-1.78] | 0.9 [95% CI 0.5-1.64] | 1.01 [95% CI 0.55-1.84] | 0.97 [95% CI 0.53-1.77] | 0.93 [95% CI 0.51-1.7] | 0.95 [95% CI 0.52-1.72] | 1.06 [95% CI 0.58-1.93] |
| Malignancy | 1.33 [95% CI 1.15-1.54] | 1.28 [95% CI 1.1-1.48] | 1.3 [95% CI 1.12-1.51] | 1.33 [95% CI 1.15-1.54] | 1.33 [95% CI 1.15-1.54] | 1.34 [95% CI 1.16-1.56] | 1.33 [95% CI 1.15-1.54] |
| Metastatic Solid Tumor | 1.92 [95% CI 1.48-2.49] | 1.78 [95% CI 1.38-2.31] | 1.94 [95% CI 1.5-2.51] | 1.92 [95% CI 1.48-2.49] | 1.91 [95% CI 1.47-2.47] | 1.93 [95% CI 1.49-2.51] | 1.86 [95% CI 1.43-2.41] |
| Myocardial Infarction | 1.13 [95% CI 0.56-2.3] | 0.97 [95% CI 0.48-1.99] | 1.12 [95% CI 0.55-2.28] | 1.14 [95% CI 0.56-2.31] | 1.12 [95% CI 0.55-2.29] | 1.15 [95% CI 0.56-2.34] | 1.08 [95% CI 0.53-2.21] |
| Mild Liver Disease | 0.96 [95% CI 0.77-1.19] | 1.12 [95% CI 0.89-1.4] | 0.95 [95% CI 0.76-1.18] | 0.98 [95% CI 0.78-1.22] | 0.92 [95% CI 0.74-1.15] | 0.98 [95% CI 0.79-1.22] | 0.99 [95% CI 0.79-1.24] |
| Moderate or Severe Liver Disease | 0.81 [95% CI 0.62-1.07] | 1.01 [95% CI 0.76-1.34] | 0.81 [95% CI 0.61-1.06] | 0.83 [95% CI 0.63-1.09] | 0.78 [95% CI 0.59-1.03] | 0.8 [95% CI 0.61-1.06] | 0.81 [95% CI 0.61-1.07] |
| Peptic Ulcer | 1.21 [95% CI 0.71-2.06] | 1.15 [95% CI 0.68-1.95] | 1.18 [95% CI 0.69-2.01] | 1.21 [95% CI 0.71-2.06] | 1.2 [95% CI 0.7-2.03] | 1.23 [95% CI 0.72-2.1] | 1.23 [95% CI 0.72-2.09] |
| Peripheral Vascular Disease | 0.92 [95% CI 0.73-1.16] | 0.84 [95% CI 0.67-1.06] | 0.92 [95% CI 0.73-1.15] | 0.92 [95% CI 0.73-1.16] | 0.91 [95% CI 0.72-1.15] | 0.91 [95% CI 0.72-1.14] | 0.91 [95% CI 0.72-1.15] |
| Renal Disease | 0.99 [95% CI 0.83-1.19] | 1.1 [95% CI 0.93-1.31] | 1 [95% CI 0.83-1.19] | 0.98 [95% CI 0.82-1.17] | 0.98 [95% CI 0.82-1.18] | 0.99 [95% CI 0.83-1.18] | 1.05 [95% CI 0.88-1.25] |
| Rheumatologic Disease | 0.65 [95% CI 0.43-1] | 0.87 [95% CI 0.57-1.32] | 0.64 [95% CI 0.42-0.99] | 0.64 [95% CI 0.42-0.98] | 0.74 [95% CI 0.48-1.13] | 0.65 [95% CI 0.42-0.99] | 0.7 [95% CI 0.46-1.07] |
| Medical Teams |  |  |  |  |  |  |  |
| Cardiology | 0.94 [95% CI 0.78-1.15] | 0.85 [95% CI 0.7-1.03] | 0.95 [95% CI 0.78-1.15] | 0.98 [95% CI 0.8-1.19] | 0.95 [95% CI 0.78-1.15] | 0.94 [95% CI 0.77-1.14] | 0.95 [95% CI 0.78-1.15] |
| Cardiac Care Unit (CCU) | 0.94 [95% CI 0.75-1.18] | 0.91 [95% CI 0.72-1.13] | 0.94 [95% CI 0.75-1.17] | 0.93 [95% CI 0.75-1.17] | 0.94 [95% CI 0.75-1.18] | 0.94 [95% CI 0.75-1.17] | 0.93 [95% CI 0.75-1.17] |
| Cardiac Care Unit-Heart Failure (CCU(HF)) | 1.41 [95% CI 1.15-1.72] | 1.49 [95% CI 1.22-1.81] | 1.4 [95% CI 1.14-1.71] | 1.48 [95% CI 1.21-1.81] | 1.44 [95% CI 1.18-1.76] | 1.52 [95% CI 1.24-1.86] | 1.41 [95% CI 1.16-1.73] |
| Cardiovascular ICU (CVICU) | 0.32 [95% CI 0.25-0.41] | 0.26 [95% CI 0.2-0.33] | 0.32 [95% CI 0.25-0.41] | 0.33 [95% CI 0.26-0.42] | 0.34 [95% CI 0.26-0.43] | 0.32 [95% CI 0.25-0.41] | 0.32 [95% CI 0.25-0.4] |
| Hematology-Oncology | 1.63 [95% CI 1.33-1.98] | 1.84 [95% CI 1.52-2.24] | 1.57 [95% CI 1.29-1.92] | 1.66 [95% CI 1.36-2.02] | 1.71 [95% CI 1.4-2.09] | 1.73 [95% CI 1.42-2.12] | 1.7 [95% CI 1.39-2.08] |
| Medicine | 1.23 [95% CI 1.01-1.5] | 1.14 [95% CI 0.94-1.4] | 1.22 [95% CI 1-1.49] | 1.26 [95% CI 1.03-1.53] | 1.25 [95% CI 1.03-1.53] | 1.25 [95% CI 1.03-1.53] | 1.19 [95% CI 0.97-1.45] |
| Medical ICU | 1.63 [95% CI 1.42-1.86] | 2.02 [95% CI 1.77-2.3] | 1.64 [95% CI 1.44-1.88] | 1.66 [95% CI 1.45-1.9] | 1.74 [95% CI 1.52-1.98] | 1.62 [95% CI 1.41-1.85] | 1.63 [95% CI 1.43-1.87] |
| Neurology | 0.72 [95% CI 0.52-0.99] | 0.63 [95% CI 0.46-0.87] | 0.72 [95% CI 0.52-0.99] | 0.73 [95% CI 0.53-1] | 0.71 [95% CI 0.52-0.98] | 0.71 [95% CI 0.52-0.98] | 0.69 [95% CI 0.5-0.95] |
| Surgical ICU | 0.77 [95% CI 0.65-0.91] | 0.79 [95% CI 0.67-0.94] | 0.77 [95% CI 0.65-0.92] | 0.77 [95% CI 0.65-0.92] | 0.8 [95% CI 0.67-0.95] | 0.77 [95% CI 0.65-0.91] | 0.75 [95% CI 0.63-0.89] |
| Surgery Specialty | 0.74 [95% CI 0.63-0.87] | 0.7 [95% CI 0.6-0.82] | 0.73 [95% CI 0.62-0.86] | 0.74 [95% CI 0.63-0.87] | 0.74 [95% CI 0.63-0.87] | 0.73 [95% CI 0.63-0.86] | 0.71 [95% CI 0.61-0.84] |
| Transplant | 0.96 [95% CI 0.79-1.16] | 1.03 [95% CI 0.85-1.25] | 0.92 [95% CI 0.76-1.11] | 0.97 [95% CI 0.8-1.17] | 0.97 [95% CI 0.8-1.17] | 0.96 [95% CI 0.79-1.16] | 1.01 [95% CI 0.84-1.23] |
| Trauma | 0.86 [95% CI 0.67-1.12] | 0.9 [95% CI 0.69-1.16] | 0.85 [95% CI 0.66-1.1] | 0.88 [95% CI 0.68-1.14] | 0.85 [95% CI 0.66-1.11] | 0.93 [95% CI 0.72-1.2] | 0.86 [95% CI 0.66-1.11] |
| Life Sustaining Treatment |  |  |  |  |  |  |  |
| Vasoactive | 1.55 [95% CI 1.31-1.84] | 2.24 [95% CI 1.9-2.64] | 1.54 [95% CI 1.3-1.82] | 1.56 [95% CI 1.32-1.85] | 1.65 [95% CI 1.39-1.95] | 1.64 [95% CI 1.39-1.94] | 1.66 [95% CI 1.4-1.96] |
| Ventilator | 0.95 [95% CI 0.81-1.11] | 1.51 [95% CI 1.3-1.75] | 0.99 [95% CI 0.84-1.16] | 0.93 [95% CI 0.79-1.09] | 0.99 [95% CI 0.84-1.16] | 0.93 [95% CI 0.79-1.09] | 0.98 [95% CI 0.84-1.15] |
| CCRT | 1.43 [95% CI 1.2-1.7] | 2.38 [95% CI 2.02-2.8] | 1.4 [95% CI 1.18-1.67] | 1.43 [95% CI 1.2-1.7] | 1.49 [95% CI 1.26-1.78] | 1.49 [95% CI 1.25-1.78] | 1.4 [95% CI 1.18-1.67] |
| Vital Signs and Lab Tests |  |  |  |  |  |  |  |
| Blood Urea Nitrogen (BUN) (mg/dl) | 1.01 [95% CI 1.01-1.01] | NA | 1.01 [95% CI 1.01-1.02] | 1.01 [95% CI 1.01-1.02] | 1.01 [95% CI 1.01-1.02] | 1.01 [95% CI 1.01-1.01] | NA |
| Bicarbonate (mEq/L) | 0.98 [95% CI 0.97-0.99] | NA | 0.98 [95% CI 0.97-0.99] | 0.98 [95% CI 0.97-0.99] | 0.98 [95% CI 0.96-0.99] | 0.98 [95% CI 0.97-0.99] | NA |
| Creatinine (CR) (mg/dl) | 0.95 [95% CI 0.9-1] | NA | 0.93 [95% CI 0.88-0.98] | 0.95 [95% CI 0.9-1.01] | 0.94 [95% CI 0.89-1] | 0.95 [95% CI 0.9-1] | NA |
| Glasgow Coma Score (GCS) | 0.87 [95% CI 0.85-0.88] | NA | 0.87 [95% CI 0.85-0.88] | 0.87 [95% CI 0.85-0.88] | 0.86 [95% CI 0.85-0.87] | 0.87 [95% CI 0.85-0.88] | 0.86 [95% CI 0.85-0.87] |
| Hematocrit (HCT) (%) | 1.02 [95% CI 1.01-1.03] | NA | 1.01 [95% CI 1-1.02] | 1.02 [95% CI 1.01-1.03] | 1.02 [95% CI 1.01-1.03] | 1.02 [95% CI 1.01-1.03] | 1.01 [95% CI 1-1.02] |
| Arterial PH (pHa) | 0.35 [95% CI 0.21-0.6] | NA | 0.31 [95% CI 0.19-0.52] | 0.42 [95% CI 0.24-0.71] | NA | 0.31 [95% CI 0.19-0.52] | 0.26 [95% CI 0.16-0.43] |
| Partial oxygen pressure (pO2a) | 1 [95% CI 1-1] | NA | 1 [95% CI 1-1] | 1 [95% CI 1-1] | NA | 1 [95% CI 1-1] | 1 [95% CI 1-1] |
| Potassium (mEq/L) | 1.04 [95% CI 0.95-1.13] | NA | 0.95 [95% CI 0.87-1.04] | 1.04 [95% CI 0.96-1.13] | 1.05 [95% CI 0.97-1.15] | 1.05 [95% CI 0.96-1.14] | NA |
| Pulse (per 10 bpm) | 1.08 [95% CI 1.06-1.09] | NA | 1.08 [95% CI 1.06-1.09] | 1.08 [95% CI 1.07-1.09] | 1.08 [95% CI 1.07-1.09] | NA | 1.08 [95% CI 1.06-1.09] |
| Respiratory Rate (breaths/min) | 1.04 [95% CI 1.03-1.05] | NA | 1.04 [95% CI 1.03-1.05] | 1.04 [95% CI 1.03-1.05] | NA | 1.04 [95% CI 1.03-1.05] | 1.04 [95% CI 1.03-1.05] |
| Systolic Blood Pressure (per 10 mmHg) | 0.93 [95% CI 0.91-0.95] | NA | 0.93 [95% CI 0.91-0.95] | 0.95 [95% CI 0.93-0.97] | 0.93 [95% CI 0.91-0.95] | NA | 0.92 [95% CI 0.9-0.94] |
| Sodium (mEq/L) | 1.01 [95% CI 1-1.02] | NA | 1 [95% CI 0.99-1.01] | 1.01 [95% CI 1-1.02] | 1.01 [95% CI 1-1.02] | 1.01 [95% CI 1-1.02] | NA |
| Total billirubin (mg/dl) | 1.04 [95% CI 1.03-1.05] | NA | 1.04 [95% CI 1.03-1.05] | 1.04 [95% CI 1.03-1.05] | 1.04 [95% CI 1.03-1.05] | 1.04 [95% CI 1.03-1.05] | 1.05 [95% CI 1.04-1.05] |
| Temperature (F) | 0.94 [95% CI 0.91-0.98] | NA | 0.93 [95% CI 0.89-0.96] | 0.95 [95% CI 0.92-0.99] | 0.95 [95% CI 0.91-0.98] | 0.97 [95% CI 0.93-1] | 0.94 [95% CI 0.91-0.97] |
| White blood cells (10^9/L) | 1.01 [95% CI 1-1.01] | NA | 1.01 [95% CI 1-1.01] | 1.01 [95% CI 1-1.01] | 1.01 [95% CI 1-1.01] | 1.01 [95% CI 1-1.01] | 1.01 [95% CI 1-1.01] |
